# Machine learning to predict early TNF inhibitor users in patients with ankylosing spondylitis

**DOI:** 10.1038/s41598-020-75352-7

**Published:** 2020-11-20

**Authors:** Seulkee Lee, Yeonghee Eun, Hyungjin Kim, Hoon-Suk Cha, Eun-Mi Koh, Jaejoon Lee

**Affiliations:** Department of Medicine, Samsung Medical Center, Sungkyunkwan University School of Medicine, 81 Irwon-ro, Gangnam-gu, Seoul, 06351 Republic of Korea

**Keywords:** Rheumatology, Spondyloarthritis, Computational biology and bioinformatics, Machine learning

## Abstract

We aim to generate an artificial neural network (ANN) model to predict early TNF inhibitor users in patients with ankylosing spondylitis. The baseline demographic and laboratory data of patients who visited Samsung Medical Center rheumatology clinic from Dec. 2003 to Sep. 2018 were analyzed. Patients were divided into two groups: early-TNF and non-early-TNF users. Machine learning models were formulated to predict the early-TNF users using the baseline data. Feature importance analysis was performed to delineate significant baseline characteristics. The numbers of early-TNF and non-early-TNF users were 90 and 505, respectively. The performance of the ANN model, based on the area under curve (AUC) for a receiver operating characteristic curve (ROC) of 0.783, was superior to logistic regression, support vector machine, random forest, and XGBoost models (for an ROC curve of 0.719, 0.699, 0.761, and 0.713, respectively) in predicting early-TNF users. Feature importance analysis revealed CRP and ESR as the top significant baseline characteristics for predicting early-TNF users. Our model displayed superior performance in predicting early-TNF users compared with logistic regression and other machine learning models. Machine learning can be a vital tool in predicting treatment response in various rheumatologic diseases.

## Introduction

Tumor necrosis factor (TNF) inhibitors are important drugs in treating patients with ankylosing spondylitis (AS)^1,2^, especially those incapable of using non-steroidal anti-inflammatory drugs (NSAIDs). Even though TNF inhibitors are useful for NSAID non-responders, they are not used as a first-line treatment for AS owing to their cost, adverse effects^3^, and increased chances of infection^4^. In Korea, medical insurance permits TNF inhibitors for patients with uncontrolled AS who have been treated with at least two kinds of NSAIDs continuously for three months^3^. However, there is insufficient NSAID treatment response in over 40% of patients^5^. Thus, NSAID non-responders inevitably suffer for months.

If we can predict the subset of patients who will need TNF inhibitors at an earlier phase, adequate treatment can be provided at an appropriate time and potential damages can be avoided. There have been reports of several factors that affect the start of TNF inhibitor use in AS^6^. However, there is no precise predictive model as to who should begin TNF inhibitors earlier than the others.

As the relationships of clinical variables with phenotypes are not linear but rather complex, newer machine learning methods outperform conventional statistical models in predictions using clinical variables^7–9^. Rheumatologic diseases are no exception. Several machine learning methods have been developed, which can predict survival^10^, disease activity ^11,12^, or drug failure^13^ in various rheumatologic diseases. These models have shown better predictive abilities compared with conventional methods. Therefore, previous studies have convinced us that machine learning will perform with greater accuracy in generating models in identifying the target population for the early use of TNF inhibitors in AS.

Herein, we present an artificial neural network (ANN)^14^ model for predicting the target population for the early use of TNF inhibitors in AS using baseline characteristics. The model combines demographic and laboratory data and identifies the subgroup that will require TNF inhibitors within six months of their diagnosis. We compare the performances of our ANN model with conventional statistical methods and other machine learning methods. We perform feature importance analysis with our best performing model to delineate the factors that are important in training the exact model. To our knowledge, this is the first attempt to generate a machine learning model to predict the early use of TNF inhibitors in patients with AS.

## Results

### Patient summary

**Figure 1:**
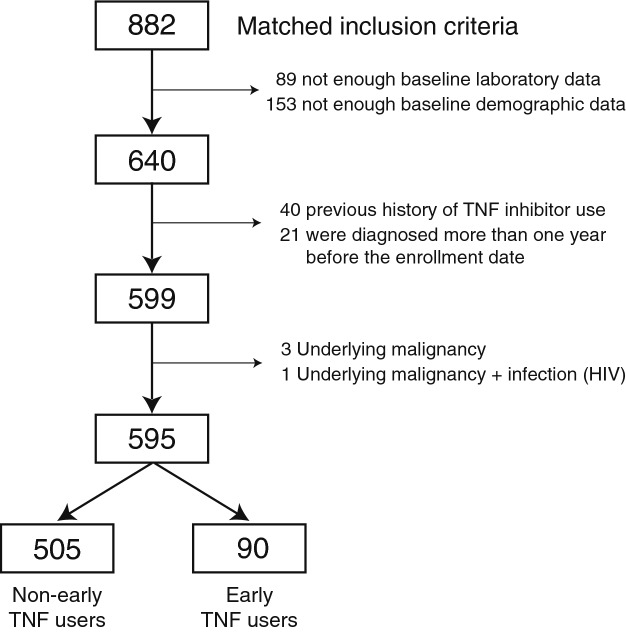
Enrollment process of this research. Eight hundred eighty-two patients matched our inclusion criteria; Eighty-nine patients did not have enough baseline laboratory data. One hundred fifty-three patients did not have at least one of demographic information. Forty patients had a previous history of TNF inhibitor use. Twenty-one patients had been diagnosed with AS at other hospitals for more than one year prior to the enrollment date. Three patients had an underlying malignancy, and one patient had an underlying malignancy and infection (HIV). Finally, five hundred ninety-five patients remained after the exclusion process. Ninety patients were designated as ‘early-TNF users; this included patients who used TNF inhibitors within six months of their diagnosis. Five hundred five patients were designated as ‘non-early-TNF users; this included patients who did not use TNF inhibitors until six months after their initial diagnosis.

The number of patients with AS who fulfilled the inclusion criteria was 882. After excluding the ineligible patients, the numbers of patients in the early-TNF and non-early-TNF user groups were 90 and 505, respectively. Figure 1 shows the enrollment process. Table 1 compares the early-TNF users and non-early-TNF users with respect to their baseline demographic characteristics and laboratory data. Demographic data were similar in both groups. The baseline laboratory results including white blood cell (WBC) count, hemoglobin (Hb), platelet count, erythrocyte sediment rate (ESR), and C-reactive protein (CRP) were significantly different. The acute-phase reactants were higher in early-TNF users.

**Table 1:** Comparing baseline characteristics of two groups: early-TNF users and non-early-TNF users.

Characteristics	Early-TNF users*	Non-early-TNF users*	*p* Value^†^
Age (years)	35.69 (13.63)	34.22 (12.81)	0.345
Female (%)	22 (24.44)	107 (21.12)	0.581
Weight (kg)	67.30 (12.81)	68.59 (14.80)	0.390
Height (cm)	166.95 (8.06)	168.63 (12.99)	0.104
WBC count	9135 (2702)	7757 (2110)	< 0.001
Hb (g/dl)	12.95 (1.79)	13.90 (1.65)	< 0.001
Platelet count (× 1000)	360.27 (102.84)	291.28 (74.76)	< 0.001
AST (IU/L)	21.76 (25.27)	21.07 (15.64)	0.803
ALT (IU/L)	23.92 (18.39)	23.59 (34.93)	0.894
BUN (mg/dl)	13.66 (4.00)	13.56 (3.83)	0.812
Creatinine (mg/dl)	0.91 (1.19)	0.86 (0.16)	0.662
ESR (mm/hr)	67.39 (35.43)	35.31 (27.68)	< 0.001
CRP (mg/dl)	3.95 (3.48)	1.41 (2.14)	< 0.001
HLA-B27 positivity (%)	88 (97.78)	495 (98.0)	> 0.999^‡^

*WBC* white blood cell, *Hb* hemoglobin, *BUN* blood urea nitrogen, *AST* aspartate aminotransferase, *ALT* alanine aminotransferase, *HLA-B27* human leukocyte antigen-B27.

*Data are presented as mean (standard deviation) unless otherwise indicated.

^†^*p* Value were obtained by t-test (continuous variables) and chi-square test (categorical variables).

^‡^*p* Value is statistically not reliable because of small samples of negative HLA-B27 in early-TNF users.

### Prediction model optimization

**Figure 2:**
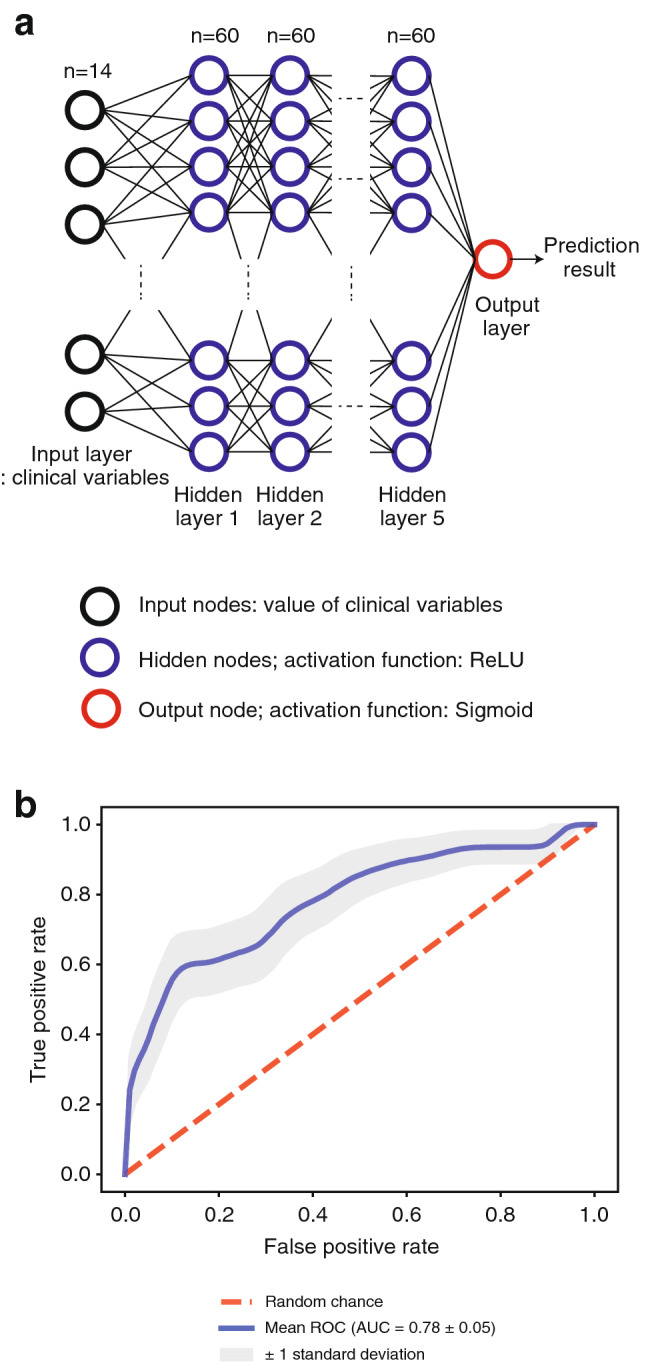
The best ANN model to predict early TNF users in patients with AS. (**a**) Architecture of the prediction model. The developed model has 5 hidden layers and 60 hidden nodes per hidden layer. The input layer has 14 nodes consisting of clinical variables. Hidden nodes are depicted as blue circles. ReLU is the activation function of all the hidden nodes. Output nodes are depicted as a red circle. The output layer has one node because we only predicted one binary variable, whether the patient was an early-TNF user or not. The sigmoid function is used as an activation function. (**b**) ROC curve of our model trained by an ANN method. The bootstrap resampling was conducted 1000 times with a standard deviation of 0.05, and the mean AUC of the results was 0.783.

We trained multiple prediction models using baseline demographics data and laboratory data through various learning methods including a conventional statistical method (logistic regression) and machine learning (support vector machine (SVM), random forest (RF), XGBoost, and ANN). The ANN, XGBoost and RF models were significantly different in terms of their detailed settings. Therefore, we trained them repeatedly by changing the architecture and hyperparameter set to achieve the best predictions. The best ANN model has five layers, except for the output layer. Each layer has 60 hidden nodes (Fig. 2a). The chosen hyperparameter set was 0.00003, 20, and 200 in the learning rate, batch size, and number of epochs, respectively. The accuracy and AUC of the ROC curve of our model were 0.878 and 0.783, respectively, in predicting early-TNF users (Fig. 2b). The F1 score of the ANN model reached higher than 95% confidence interval for a random prediction when the models had 60 hidden nodes with 5–10 hidden layers. F1 score decreased when the number of hidden nodes was above 60. With 60 hidden nodes, the AUC of the ROC curve decreased from 5 to 10 hidden layers (Supplementary Fig. S1a–b). In this setting, 0.00003 was the most appropriate value for the learning rate (Supplementary Fig. S1c–d). This results showed that more complex models do not necessarily yield better performances. In addition, ANN models, which have similar architectures and hyperparameters, provide comparable performances with the model with the best characteristics, indicating the robustness of our ANN method. We applied a similar process with the XGBoost and RF models (Supplementary Fig. S1e–l). Hyperparameter set for the maximum depth of a tree, learning rate, and gamma of the XGBoost were 9, 0.1, and 1, respectively. And the maximum depth of a tree, number of trees, minimum sample split, and minimum leaf samples of the RF were none (no limit), 30, 1, and 2. In the sensitivity analysis, we observed robust performances in predicting early-TNF users by changing methods to divide the test dataset (AUC = 0.766 without the cross-validation method, and AUC = 0.791 with the independent test dataset divided in advance, Supplementary Fig. S2).

### Performance of various prediction models

**Figure 3:**
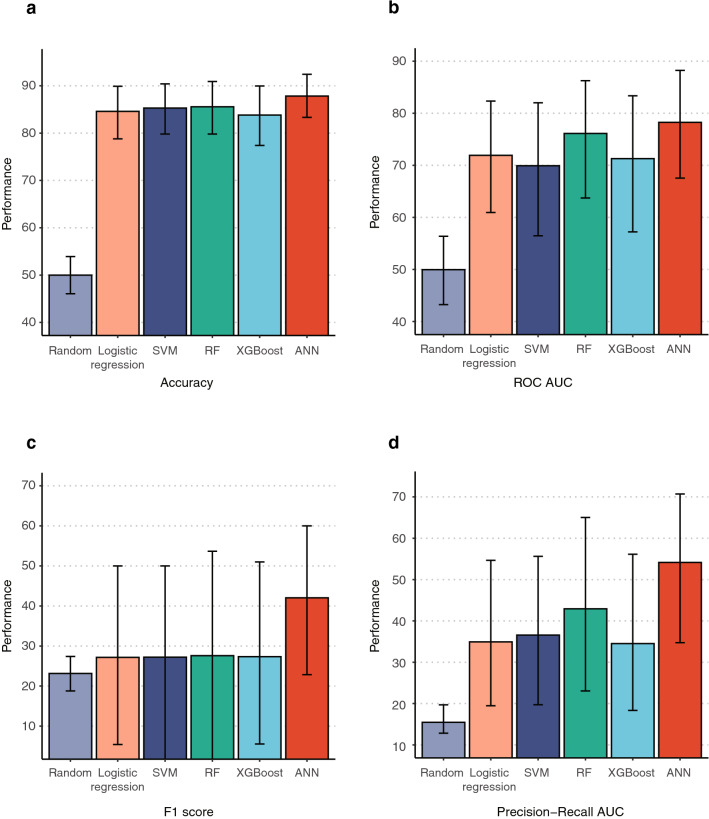
Performances of models trained by various methods (random prediction, logistic regression, SVM, RF, XGBoost, and ANN): (**a**) by accuracy, (**b**) by the AUC of the ROC curve from the sensitivity–specificity plot, (**c**) by F1 score, (**d**) by the AUC of the precision-recall curve.

We checked the accuracy, AUC of the ROC curve, F1 score, and AUC of the precision-recall curve to evaluate the performances of various models**.** The accuracies of the logistic regression, SVM, RF, XGBoost, and ANN models were 0.846, 0.853, 0.856, 0.838, and 0.878, the AUCs of the ROC curve of sensitivity and specificity were 0.719, 0.699, 0.761, 0.713, and 0.783, the F1 scores were 0.272, 0.272, 0.276, 0.274, and 0.420, and the AUCs of the precision-recall curve were 0.349, 0.366, 0.429, 0.345, and 0.541, respectively (Fig. 3). Among these methods, ANN showed the best performance in predicting early-TNF users. The better performance of the ANN model compared to the logistic regression model suggests that the relationship between clinical information and the predictive outcome is nonlinear.

### F1 score and the AUC of the precision-recall curve with balanced test dataset

Although the F1 score and the AUC of the precision-recall curve of the ANN model were better than those of other methods and a random prediction, the performance were low numerically. The main reason for the low performance was the use of an imbalanced dataset. Thus, we checked the F1 score and AUC of the precision-recall curve using a balanced test dataset, which showed comparable performances in terms of the accuracy and AUC of the ROC curve (Supplementary Fig. S3).

### Feature importance of the trained model

**Figure 4:**
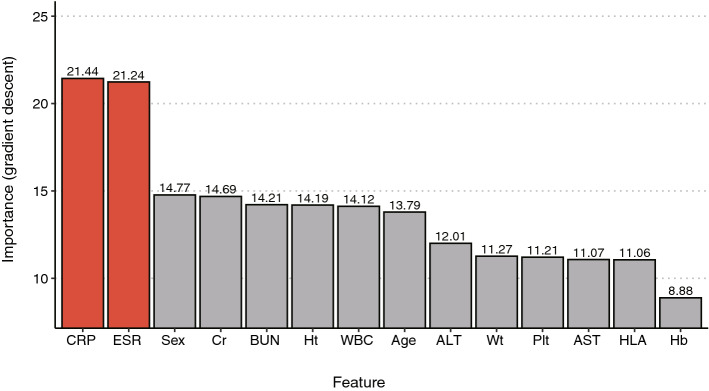
The result of feature importance analysis from our model trained by an ANN method. The x-axis shows the input clinical variables. The y-axis represents the feature importance score calculated by the ‘risk backpropagation’ method. The two most important features are highlighted in red. *Ht* height, *Plt* platelet, *HLA* HLA-B27, *Wt* weight, *Cr* creatinine, *Hb* hemoglobin.

The ‘black box’ nature of neural networks is a critical barrier to analyze the features that are used in training neural network models^15^. Several methods have been suggested to evaluate feature importance despite this limitation. We calculated feature importance scores by a ‘risk backpropagation’ method (See “Methods”)^16^. The top two important features to train the prediction model were the CRP and ESR levels (Fig. 4). We also evaluated feature importance by XGBoost and RF, and results showed that CRP and ESR ranked 1st and 2nd places, respectively (Supplementary Fig. S4). Other features showed inconsistent results when different machine learning methods were applied.

## Discussion

We presented a machine-learning model to determine which AS patients will use TNF inhibitors in the early onset of the disease. To the best of our knowledge, this is the first attempt in this regard that has used machine learning. We have shown that the model obtained through an ANN can predict early TNF inhibitor users more accurately than conventional statistical models. Furthermore, the ANN model could suggest the most influential factors about the correlation between baseline clinical data and outcome by the feature importance analysis of each clinical variable.

Our most successful ANN model has 5 hidden layers and 60 hidden nodes in each hidden layer. We tested multiple models with various architectures, as there is no consensus regarding the optimal architecture and hyperparameter set when trying to generate a neural network model in advance. The architecture of our model is decided by the input data itself. The number of hidden nodes for the best performance is correlated with the number of our input data variables. Models with too many hidden nodes (e.g. 100) compared with the number of input variables (14, in this research) did not show better performances than simpler models. The number of hidden layers of best performance, i.e., five, implies that the correlation between the input data (baseline clinical data of patients) and output data (early TNF inhibitor use) is non-linear.

We tried various machine learning methods including SVM, RF, XGBoost, and ANN. SVM^17,18^ is relatively older than the other methods but has a suitable performance in simple image discrimination and little computational burdens. RF and XGBoost are newer methods, and are both ensemble models based on decision trees. RF consists of numerous smaller decision trees^19^, and XGBoost is a gradient boosting method. These approaches have great powers to divide groups and include feature importance analysis. However, ANN shows a better performance than RF or XGBoost in many regards^20,21^. In our data, only ANN outperformed the conventional statistical model and logistic regression. This may be due to the complex relationship between clinical inputs and outputs.

Prediction models can also give us insight into baseline clinical variables. Many machine learning tools have distinctive methods to analyze the importance of input variables^22,23^. However, neural networks have notorious ‘black box’ characteristics; these refer to the difficulty in analyzing feature importance. Several methods that overcome this limitation have recently been announced^15,16^. The important variables derived from the ‘risk backpropagation’ method with our ANN model were CRP and ESR. The top two variables were equal to the feature importance analysis in both the RF and XGBoost models. In addition to these two variables, the WBC count, Hb, and platelet count were significantly different between early-TNF users and non-early-TNF users. Thus, we cannot distinguish which variables are more for dividing the two groups when applying a conventional statistical method. As shown in this example, it would be possible to identify relatively more important factors to divide the two groups using the machine learning method that cannot be distinguished through a conventional statistical analysis.

Our approach, however, has some limitations. First, we could not add information concerning a review of the system and physical examinations. Due to the limitation of retrospective studies, part of the clinical information and physical examination data were missing for some patients. Therefore, we did not use the frequently missing information. Instead, we showed that it is possible to generate a model with acceptable performances using only laboratory results and demographic data recorded in a regular clinical setting. Second, we only used patient information from one hospital. To reduce the bias as much as possible, we implemented a cross-validation method and repeated it three times. Still, part of our model might be informed about the bias of training the cohort.

In conclusion, we made an ANN model that could predict early TNF users in patients with AS using baseline clinical data. It has 5 hidden layers and 60 hidden nodes within each layer. The model has a better performance than a logistic regression model and the other machine learning models (SVM, RF, and XGBoost). Feature important analysis showed that CRP and ESR were the most important input variables to distinguish early TNF users.

## Methods

### Study design and patient demographics

This was a retrospective longitudinal study conducted at Samsung Medical Center (SMC, Seoul, Republic of Korea). Our target cohort population was the patients (1) who fulfilled the modified New York criteria for ankylosing spondylitis (AS) or the assessment of spondyloarthritis international society (ASAS) axial spondyloarthritis (SpA) criteria who started a follow up at the rheumatology department of SMC between Dec 2003, and Sep 2018, and (2) who had followed up more than three months. In order to find this cohort in the electric health database, we included patients who (1) had attended the rheumatology outpatient clinic of the SMC with at least two registered visit that were more than three months apart, (2) had received any ICD9 and/or ICD10 codes for AS by their usual rheumatologist in at least in two consecutive visits, and (3) were diagnosed with AS between Dec 1, 2003, and Sep 1, 2018. We excluded patients who did not have a complete set of baseline laboratory results or baseline demographic data (weight and height); were diagnosed one year or more before their first visit to SMC; had an underlying malignancy, infection, or other rheumatologic diseases; were pregnancy, and who had previous experiences with TNF inhibitors before the index date. The definition of the index date will be explained in the clinical data preparation section.

### Clinical data

We gathered baseline clinical data including age, sex, height, weight, baseline laboratory results, and HLA-B27. Baseline laboratory results consisted of white blood cell count, hemoglobin, platelet count, blood urea nitrogen (BUN), creatinine, aspartate transaminase (AST), alanine transaminase (ALT), ESR, and CRP. The laboratory results with the closest initial diagnosis date were chosen as baseline laboratory results. We defined the date in which the baseline laboratory tests were performed as the index date. The clinical data was used as machine learning features. The patients were divided into two groups, ‘early-TNF users’ and ‘non-early-TNF users’; those who had used TNF inhibitors within six months and who did not use TNF inhibitors until six months after the baseline laboratory tests, respectively. Non-early-TNF users include patients who used TNF inhibitors six months after their diagnosis.

### Model design

Using a clinical dataset matrix, we trained the prediction models to distinguish between early-TNF users and non-early-TNF users. There is no previous knowledge concerning model architecture and hyperparameter sets that are suitable to predict clinical prognosis using electric health records. Therefore, we tested multiple machine learning models by varying the architecture and hyperparameters. Model architecture for the ANN includes the number of layers and hidden nodes; hyperparameters include learning rate, batch size, and the number of epochs. In the case of XGBoost, the hyperparameters include the learning rate, maximum depth of a tree, and the gamma value. For the RF model, the hyperparameters include the maximum depth of a tree, total number of trees, minimum sample split, and minimum leaf samples. The learning rate is the number of changes newly acquired information undergoes in overriding old information, batch size refers to the number of training examples utilized in a single iteration, epochs refer to one forward pass and one backward pass of all the training datasets, gamma refers to minimum loss reduction required to make a further partition on a leaf node of the tree, the minimum sample split refers to the minimum number of samples required to split an internal node, and the minimum leaf samples refers to the minimum number of samples required to be at a leaf node. Detailed learning methods and background mathematics are covered in Supplementary Information.

### Performance evaluation

The prediction models were evaluated in three rounds of three-fold cross-validation^24^. As the early TNF users are not equally distributed in the dataset, we used stratified cross-validation to divide the dataset. In each round, an entire dataset was randomly and equally divided into three, with stratified probability. Two of these parts were used as the training dataset, and the final part was used as the test dataset. The process was repeated three times. A model was trained using the training dataset and scored on the other; this process was repeated after swapping the test datasets. Three rounds of the three-fold cross-validation gave a total of 9 scores, the average of which became the estimated performance of the model. The performances were compared by the AUC of the ROC curve, accuracy, F1 score, and AUC of the precision-recall curve. For sensitivity analysis, we tried various methods for dividing the test dataset. First, we simply divided the dataset into three groups: training, validation, and test dataset rather using cross-validation. A model was trained using the training and validation datasets, and the AUC of the ROC curve was assessed using the test dataset. Second, we divided the independent test dataset before generating the model and the prediction model using the rest of the data. We evaluated the performance of the model using the independent test dataset again to compensate for the lack of independent cohort data. Additional information for performance evaluation is included in the Supplementary Information.

### Comparison with other methods

We compared our ANN model with other models trained by other machine learning methods and a conventional statistical method. We chose SVM, RF, and XGBoost as comparing machine learning methods because they are not only popular but are representative of supervised learning methods other than neural networks. A logistic regression model was chosen as representative of the conventional statistical method. We performed three rounds of three-fold cross-validation on these models identical to that of our ANN model. The logistic regression model was trained and scored by three-fold cross-validation to evaluate its performance but it did not need a three-rounds learning procedure because of its lack of hyperparameters.

### Feature importance analysis

One of the disadvantages of ANN model is its ‘black box’ characteristics, which are indicative of the difficulty in identifying the importance of each feature used in the model’s training. There are several methods to evaluate feature importance despite this limitation^15,16^. We used the differential value of the prediction score in changing each input variable for feature importance. In previous research, this method is called ‘risk backpropagation’^16^.

$$Feature\ importanc{e}_{i}= \frac{\partial \ prediction \ score}{\partial \ inpu{t}_{i}}$$

where, $inpu{t}_{i}$ = (value of *i*th variable).

The more important the role an input variable plays in model training, the more the output value (in this case, the prediction score) will be changed as the input changes; i.e., when the output value is differentiated into a specific input variable, the larger the value of the differential, the more important the variable is in training the prediction model. We calculated the differential value of each input variable to reveal the feature importance. In addition, we performed feature importance analysis by XGBoost and RF to verify the robustness of the results. The method used to find feature importance by XGBoost and RF is explained in Supplementary Information.

### Statistical analysis

Python (ver. 3.7.3) and R (ver. 3.6.3)^25^ were used for statistical analysis. We used Keras (ver. 2.2.4)^26^ and TensorFlow (ver. 1.14)^27^ to construct ANN models. ‘Scikit-learn’ module^28^ was used for the logistic regression, SVM, RF, and XGBoost models, and for calculating the performances. A ROC graph was depicted by the ‘matplotlib’ module^29^ in Python. Figures were depicted using ‘ggplot2 2.2.1’ package^30^.

### Ethics approval

Data was extracted from the Clinical Data Warehouse Darwin-C of Samsung Medical Center for this study. This study was approved by the Institutional Review Board of the Samsung Medical Center, Seoul, South Korea (IRB No.: 2019-04-079) and the informed consent requirement was waived by the IRB, because the study information was de-identified. All methods were carried out in accordance with relevant guideline and regulations.

## Supplementary information

Supplementary Information

## References

[1] Braun J (2002). Treatment of active ankylosing spondylitis with infliximab: a randomised controlled multicentre trial. Lancet.

[2] Gorman JD, Sack KE, Davis JC (2002). Treatment of ankylosing spondylitis by inhibition of tumor necrosis factor alpha. N. Engl. J. Med..

[3] Ma Z (2017). Safety of tumor necrosis factor-alpha inhibitors for treatment of ankylosing spondylitis: a meta-analysis. Medicine (Baltimore).

[4] Komaki Y (2017). Efficacy, safety and pharmacokinetics of biosimilars of anti-tumor necrosis factor-alpha agents in rheumatic diseases; A systematic review and meta-analysis. J. Autoimmun..

[5] Baraliakos X (2017). Efficiency of treatment with non-steroidal anti-inflammatory drugs according to current recommendations in patients with radiographic and non-radiographic axial spondyloarthritis. Rheumatology (Oxford).

[6] Pham T (2006). An international study on starting tumour necrosis factor-blocking agents in ankylosing spondylitis. Ann. Rheum. Dis..

[7] Kakadiaris IA (2018). Machine learning outperforms ACC/AHA CVD risk calculator in MESA. J. Am. Heart Assoc..

[8] Steele AJ, Denaxas SC, Shah AD, Hemingway H, Luscombe NM (2018). Machine learning models in electronic health records can outperform conventional survival models for predicting patient mortality in coronary artery disease. PLoS ONE.

[9] Wu J, Roy J, Stewart WF (2010). Prediction modeling using EHR data: challenges, strategies, and a comparison of machine learning approaches. Med. Care.

[10] Lezcano-Valverde JM (2017). Development and validation of a multivariate predictive model for rheumatoid arthritis mortality using a machine learning approach. Sci. Rep..

[11] Ceccarelli F (2017). Prediction of chronic damage in systemic lupus erythematosus by using machine-learning models. PLoS ONE.

[12] Norgeot B (2019). Assessment of a deep learning model based on wlectronic health record data to forecast clinical outcomes in patients with rheumatoid arthritis. JAMA Netw. Open.

[13] Guan Y (2019). Machine learning to predict anti-tumor necrosis factor drug responses of rheumatoid arthritis patients by integrating clinical and genetic markers. Arthritis Rheumatol..

[14] Singh G (1996). Gastrointestinal tract complications of nonsteroidal anti-inflammatory drug treatment in rheumatoid arthritis. A prospective observational cohort study. Arch. Intern. Med..

[15] Shrikumar, A., Greenside, P. & Kundaje, A. Learning important features through propagating activation differences. In *Proceedings of the 34th International Conference on Machine Learning* **70**, 3145–3153 (2017).

[16] Yousefi S (2017). Predicting clinical outcomes from large scale cancer genomic profiles with deep survival models. Sci. Rep..

[17] Hearst MA (1998). Support vector machines. IEEE Intell. Syst. Appl..

[18] Burges CJC (1998). A tutorial on support vector machines for pattern recognition. Data Min. Knowl. Discov..

[19] Breiman L (2001). Random forests. Mach. Learn..

[20] Ahmad MW, Mourshed M, Rezgui Y (2017). Trees vs neurons: comparison between random forest and ANN for high-resolution prediction of building energy consumption. Energy Build..

[21] Burges CJC (1998). A tutorial on support vector machines for pattern recognition. Data Min. Knowl. Discov..

[22] Genuer R, Poggi JM, Tuleau-Malot C (2010). Variable selection using random forests. Pattern Recognit. Lett..

[23] Weston, J. *et al.* Feature selection for SVMs. In *Proceedings of the 13th International Conference on Neural Information Processing Systems* 647–653 (2000).

[24] Kim JH (2009). Estimating classification error rate: repeated cross-validation, repeated hold-out and bootstrap. Comput. Stat. Data Anal..

[25] R Core Team. *R: a language and environment for statistical computing*. R Foundation for Statistical Computing, Vienna, Austria. https://www.R-project.org/ (2020).

[26] Chollet, F. *et al*. Keras. https://keras.io (2015).

[27] Abadi, M. *et al.* Tensorflow: a system for large-scale machine learning. In *Proceedings of the 12th USENIX Conference on Operating Systems Design and Implementation* 265–283 (2016).

[28] Pedregosa F (2011). Scikit-learn: machine learning in python. J. Mach. Learn. Res..

[29] Hunter JD (2007). Matplotlib: a 2D graphics environment. Comput. Sci. Eng..

[30] Wickham H (2016). ggplot2: Elegant Graphics for Data Analysis.

