# Supplementary material for: Machine learning to predict early TNF inhibitor users in patients with ankylosing spondylitis
Source: Sci Rep. 2020 Nov 20;10:20299. doi: 10.1038/s41598-020-75352-7 (PMC7679386; doi:10.1038/s41598-020-75352-7)
Supplement: Supplementary file 1 — Supplementary Information [file 41598_2020_75352_MOESM1_ESM.pdf]

Supplementary Information

Machine learning to predict early TNF inhibitor users in patients with ankylosing spondylitis

Seulkee Lee, Yeonghee Eun, Hyungjin Kim, Hoon-Suk Cha, Eun-Mi Koh, and Jaejoon Lee

Department of Medicine, Samsung Medical Center, Sungkyunkwan University School of Medicine, Seoul, Republic of Korea

Supplementary Information

Model design: Artificial Neural Network (ANN)

The developed artificial neural network model consists of an input layer, two hidden layers, and an output layer.

1) Input layer to the first hidden layer

The input layer takes an input feature vector (X^y) of size M, which is the total number of input variables. The number of individual input datasets is represented by y. Each input dataset has a different scale, continuous variables that are standardized to a mean of zero, and two standard deviations. Other categorical variables such as sex and HLA-B27 can be used directly.

The initial value of each weight is to be chosen randomly between -0.3 and 0.3. The first feature map array (I^1) is obtained with an input vector and tunable vector of weights (w^1), as follows:

$$I^1_{n_1} = \sum_{m=1}^{M} w^1_{mn_1} \times X^y_m$$

$$\text{where } 1 \leq n_x \leq N_x, N_x = (\text{total number of nodes of the } x - \text{th hidden layer})$$

Here, $w^1_{mn_1}$ is the weight factor of the input layer to the first hidden layer, m-th is the input feature, and n_1-th is the hidden node; $I^1_{n_1}$ is the input feature map array of n_1-th hidden nodes. We added a bias vector, b_{n_1}, and then applied a rectified linear unit (ReLU) as an activation function of each node.

$$O^1_{n_1} = \text{ReLU}(I^1_{n_1} + b_{n_1}),$$

$$\text{where ReLU(x)} = \begin{cases} x \text{ if } x \geq 0 \\ 0 \text{ if } x < 0 \end{cases}$$

2) Between the hidden layers

The output data of the previous hidden layer is used as input data for the next hidden layer. With the output vector of the previous hidden layer ($O^{k-1}_{n_{k-1}}$) and tunable vector of weights (w^k), the k-th feature map array (I^k) is obtained as follows:

$$I^k_{n_k} = \sum_{n_{k-1}=1}^{N_{k-1}} w^k_{n_{k-1}n_k} \times O^{k-1}_{n_{k-1}}$$

$$\text{where } 2 \leq k \leq K + 1, K = \text{(total number of hidden layers)}$$

Here, $w^{k}_{n_{k-1}n_k}$ is the weight factor of the input layer to the *k*-th hidden layer, n_{k-1}-th the output node of the (*k*–1)-th hidden layer, and n_k-th the hidden node of *k*-th hidden layer; $I^{k}_{n_k}$ is the input feature map array of n_k-th hidden node at *k*-th hidden layers. We added a bias vector, b_{n_k}, and then applied ReLU as an activation function for each node.

$$O^{k}_{n_k} = \text{ReLU}\left(I^{k}_{n_k} + b_{n_k}\right),$$

$$\text{where ReLU(x)} = \begin{cases} x \text{ if } x \geq 0 \\ 0 \text{ if } x < 0 \end{cases}$$

We use ReLU as an activation function in all hidden nodes.

3) Output layer

At the last layer of our ANN model, we connect to a sigmoid function, which is frequently used for binary output data.

$$p = \sum_{n_K=1}^{N_K} w^{K}_{n_K n_{K+1}} \times O^{K}_{n_K}$$

$$p' = \frac{1}{1 + e^{-p}} \text{ (sigmoid function)}$$

Here $w^{K}_{n_K n_{K+1}}$ is the weight factor of the output layers. We can obtain the input value of the output layer as the sum of all weights from the last hidden layer, *p*. At the output layer, we perform the sigmoid function rather than ReLU because it has a better performance for the output layer of binary results. Therefore, *p'* is the final predictive value for each category in the ANN model.

Model training

The input data for the model is split into three same size datasets. Each patient data is randomly allocated to three different datasets. The divided input dataset is used as a training, validation, and testing set, respectively. We change the role of the divided dataset after the initial training, which is called cross-validation.

The value of the hyperparameter set is to be chosen randomly. The validation and testing sets are used to select the best hyperparameter set and report the final performance levels, respectively. We train the

model weights to minimize the negative log likelihood (NLL), which is defined as follows:

$$\mathrm{NLL}(\theta) = -\frac{1}{B}\sum_{s=1}^{B}(Y\log f(\theta)^{s} + (1 - Y)\log(1 - f(\theta)^{s})),$$

where $f(\theta)^{s}$ is an output for the clinical dataset of s-th patient in a mini-batch of size B from the training set. Each target, Y, serves as the dependent variable and can be either 1 (for true cases or patients who used the TNF alpha inhibitor within six months from their baseline) or 0 (for false cases or patients who did not use the TNF alpha inhibitor until six months from their baseline). The NLL is composed of parameters (θ) including w_{nk}^{k} and b_k, which are updated by a standard backpropagation algorithm with momentum.

Performance evaluation

In case of the limited amount of data, dividing the dataset into training, validation, and test can be important. As we use increase the amount of data as the test dataset, the training dataset shrinks. It leads to the model of decreasing performance. If we use a small amount of data as the test dataset, the performance may be checked highly by chance when the test dataset fits well and randomly with the machine learning model. To avoid such problems, cross-validation is a popular method to check the model performance (1). The k-fold cross-validation refers to when the entire dataset is randomly and equally divided into the number of k. If the dataset has an unbalanced feature distribution, stratified cross-validation can be used for ensuring the similar distribution of each dataset. Cross-validation used all data as a test dataset at least once. It guarantees that the model is sufficiently robust, providing a good performance with any part of the dataset. Additionally, we checked the performances without using cross-validation for sensitivity analysis. We divided our dataset into three parts: training, validation, and test datasets by the 6:2:2 ratio. Same as the cross-validation, we divided each dataset using stratified random sampling. Finally, we left the independent test dataset before the whole generating process of the model. Ideally, the independent data from another cohort are best for proving whether the model performs well in general circumstances. Although we had the only cohort data, part of the dataset was divided in advance similar to an independent cohort. The rest of data were used in training, validation, and the test process. After finishing the model generating process, we assessed the performance of the model using an independent test dataset, which was made before the entire course. For this work, we divided data into

data for model generation and independent test dataset by a 8:2 ratio. Data for model generation were divided again into training, validation, and the test dataset by 6:2:2 for the subsequent processes. All stratified cross-validation and stratified sampling processes were performed using the 'Scikit-learn' module in Python (2).

Confidence interval of prediction performance

We used bootstrap to calculate confidence interval of the prediction performance (3). We used 1000 bootstrap iterations by sampling with a replacement using the *resample()* function from the 'Scikit-learn' module in Python. Using each bootstrap dataset, the prediction model was trained and evaluated in terms of the prediction performance. Therefore, we obtained 1000 performances of each machine learning algorithms. For a confidence interval of 95%, we selected the value at the 2.5 percentile as the lower bound and the 97.5 percentile as the upper bound.

Performance of random prediction

To compare the performances of various prediction models with random prediction, we prepared a dummy test dataset for an evaluation. We generated a prediction dataset consisting of an equal number of true and false values from the real dataset. The prediction values (true or false) were randomly allocated for each individual. We checked the performances (accuracy, AUC of ROC curve, F1 score, and AUC of the precision-recall curve) of each random dataset. We repeated the process 1000 times and reported the mean and 95% confidence interval of the performances.

Feature importance by RF and XGBoost

In order to verify that the feature importance that was derived by our ANN can be reproduced by other machine learning methods, we performed feature importance analysis by RF with an identical dataset. After training the RF model as described above, we calculated the 'mean decrease accuracy', which is defined as the average of the total decrease in accuracy when imputing each input feature dataset. If a feature plays an important role in training an RF model, the imputed input feature dataset results in a lower performance model and the 'mean decrease accuracy' value will increase. The implementation of this aspect was performed as a function of the feature_importances of the 'Scikit-learn.ensemble' module

in Python. Similarly, we performed a feature importance analysis using XGBoost. The ‘xgboost’ module in Python functions similarly to the feature_importances, and calculates the relative feature importance of each feature.

1. Kohavi R. A study of cross-validation and bootstrap for accuracy estimation and model selection. Proceedings of the 14th international joint conference on Artificial intelligence - Volume 2; Montreal, Quebec, Canada. 1643047: Morgan Kaufmann Publishers Inc.; 1995. p. 1137-43.
2. Pedregosa F, Ga, #235, Varoquaux l, Gramfort A, Michel V, et al. Scikit-learn: Machine Learning in Python. J Mach Learn Res. 2011;12:2825-30.
3. Raschka S. Model evaluation, model selection, and algorithm selection in machine learning. arXiv preprint arXiv:181112808. 2018.

Supplementary Fig. S1

a

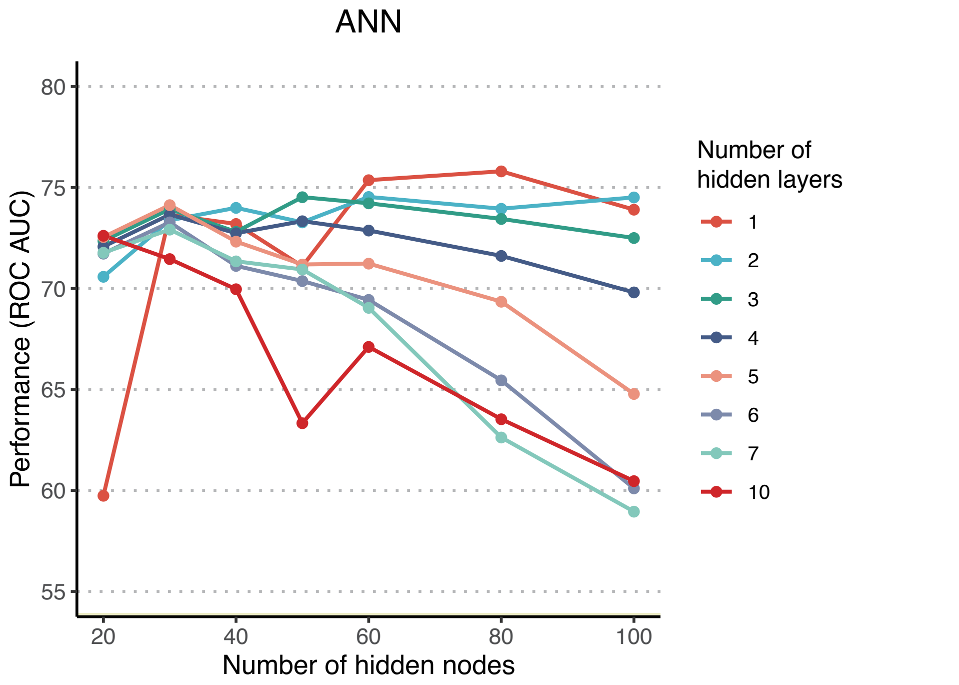

b

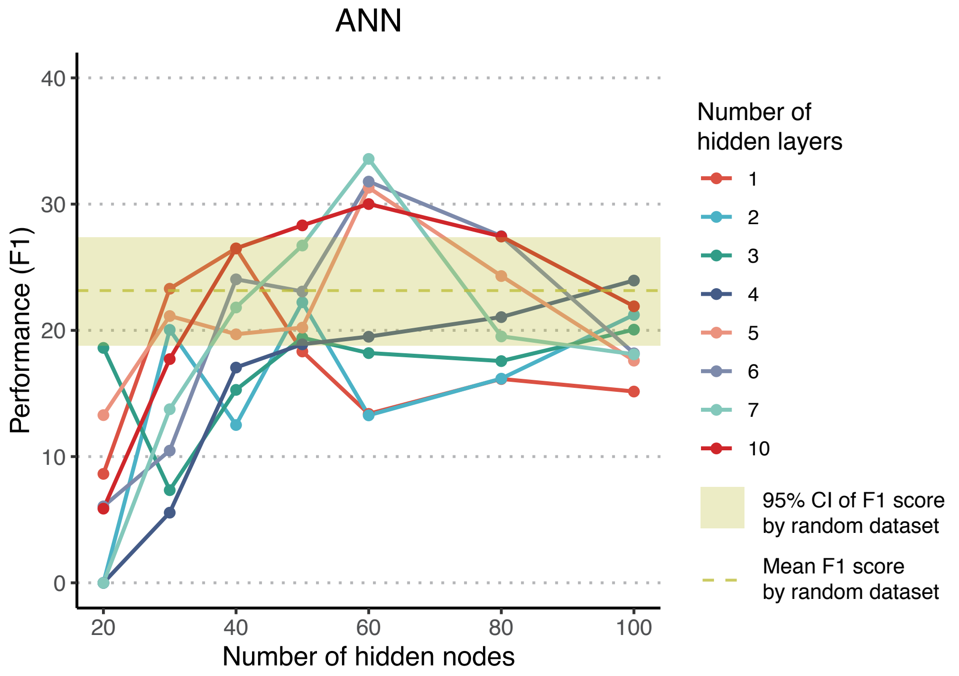

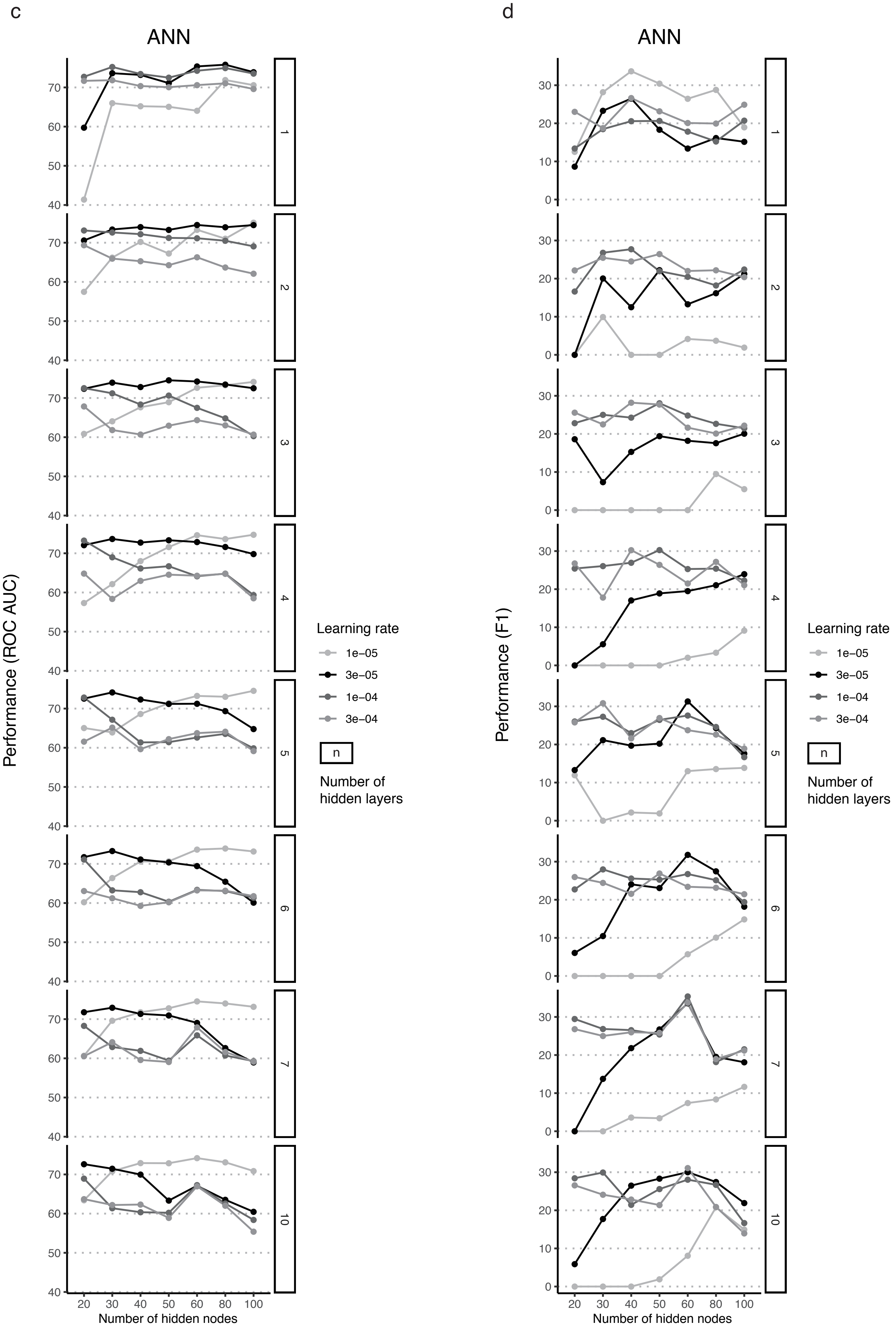
c
ANN
Performance (ROC AUC)
Number of hidden nodes
Learning rate
1e−05
3e−05
1e−04
3e−04
n
Number of hidden layers
d
ANN
Performance (F1)
Number of hidden nodes
Learning rate
1e−05
3e−05
1e−04
3e−04
n
Number of hidden layers

e

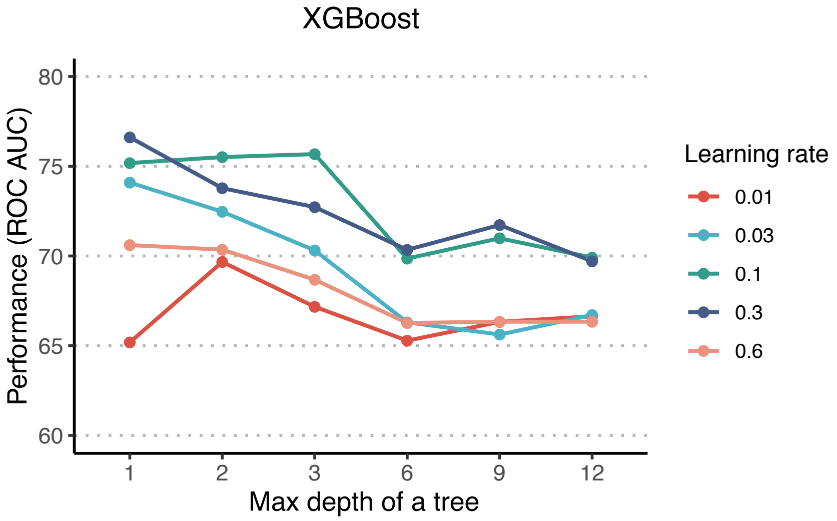
XGBoost
Performance (ROC AUC)
80
75
70
65
60
1
2
3
6
9
12
Max depth of a tree
Learning rate
0.01
0.03
0.1
0.3
0.6

f

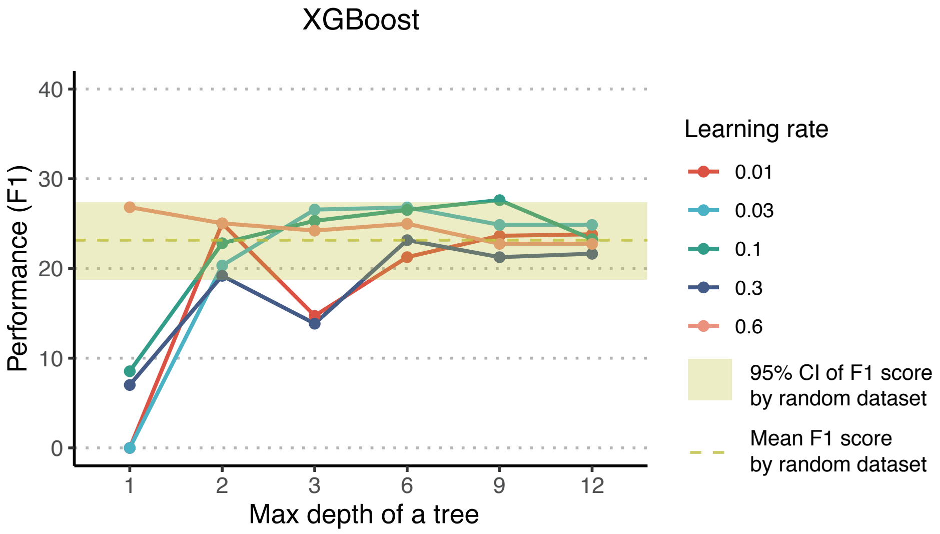
XGBoost
Performance (F1)
40
30
20
10
0
1
2
3
6
9
12
Max depth of a tree
Learning rate
0.01
0.03
0.1
0.3
0.6
95% CI of F1 score by random dataset
Mean F1 score by random dataset

g

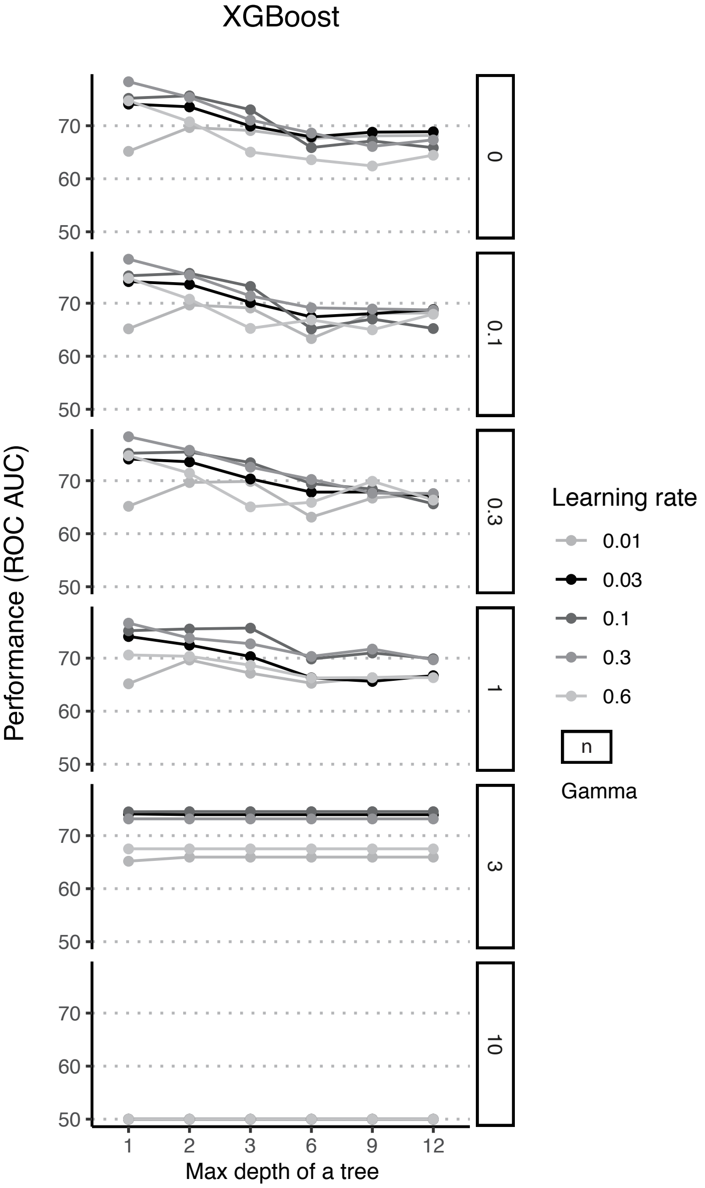
XGBoost
Performance (ROC AUC)
Max depth of a tree
Learning rate
0.01
0.03
0.1
0.3
0.6
n
Gamma
0
0.1
0.3
1
3
10

h

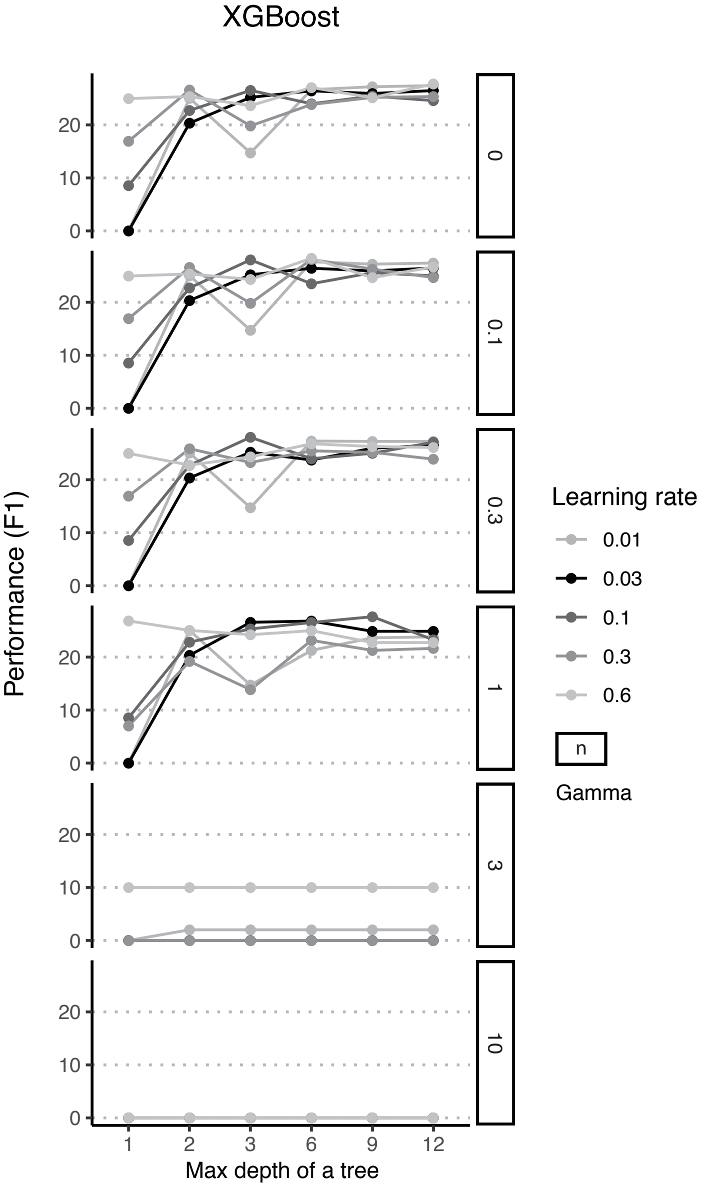
XGBoost
Performance (F1)
Max depth of a tree
Learning rate
0.01
0.03
0.1
0.3
0.6
n
Gamma
0
0.1
0.3
1
3
10

i

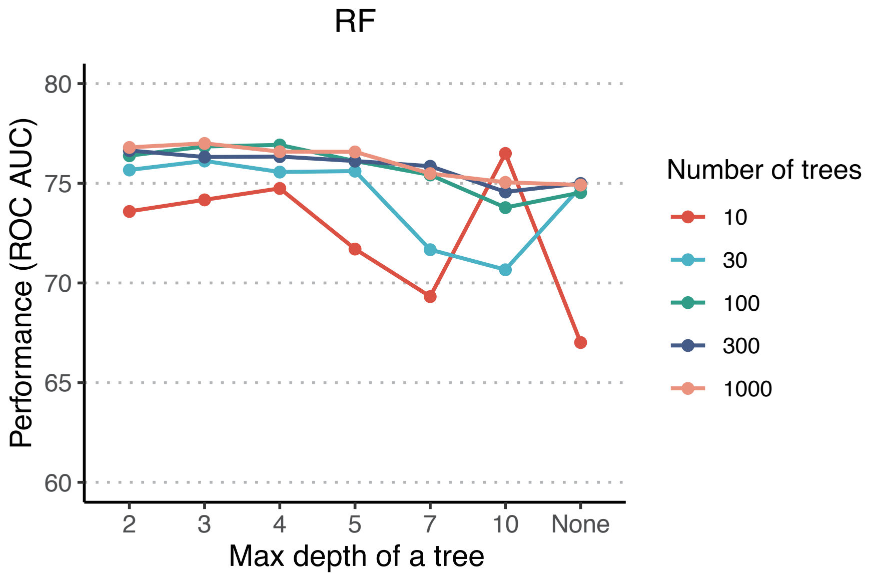

RF
Performance (ROC AUC)
80
75
70
65
60
2
3
4
5
7
10
None
Max depth of a tree
Number of trees
10
30
100
300
1000

j

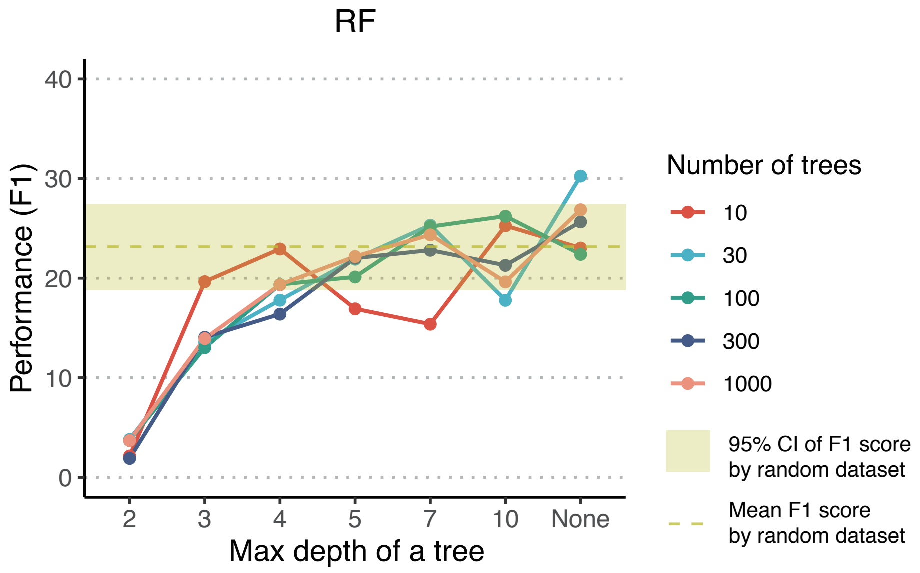

RF
Performance (F1)
40
30
20
10
0
2
3
4
5
7
10
None
Max depth of a tree
Number of trees
10
30
100
300
1000
95% CI of F1 score
by random dataset
Mean F1 score
by random dataset

k

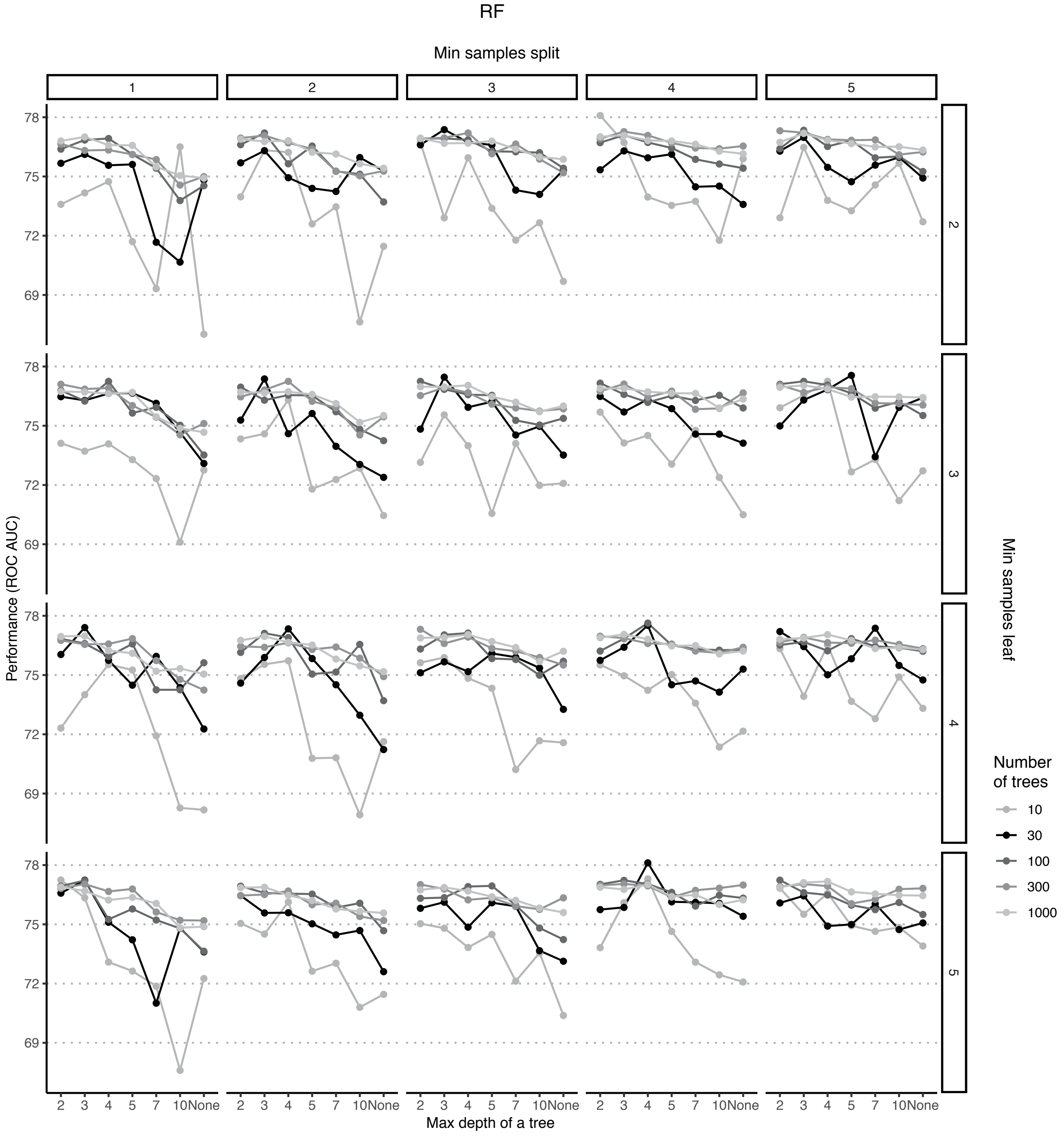
RF
Min samples split
1
2
3
4
5
Min samples leaf
2
3
4
5
Performance (ROC AUC)
78
75
72
69
Max depth of a tree
2
3
4
5
7
10
None
Number of trees
10
30
100
300
1000

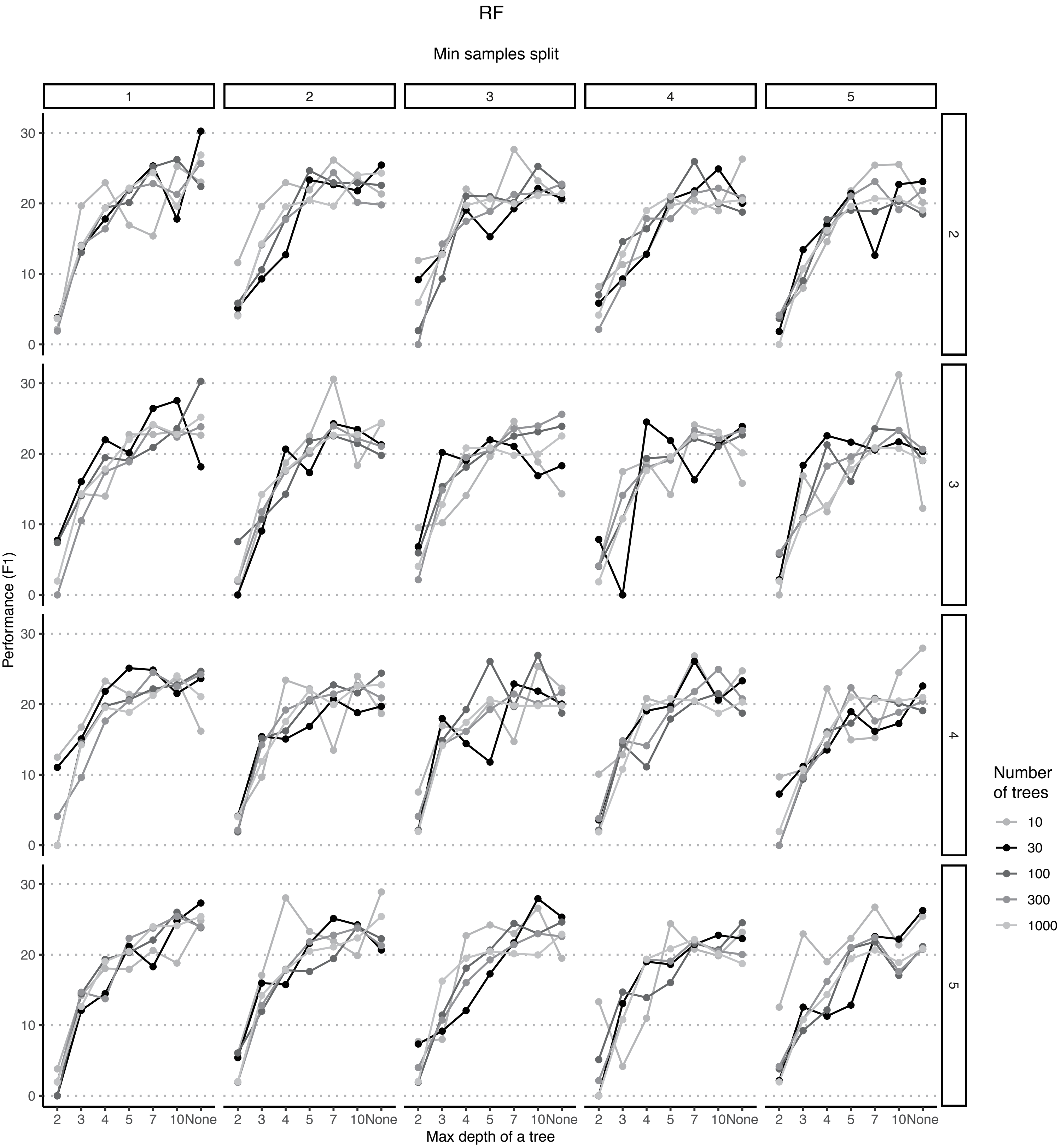
RF
Min samples split
1
2
3
4
5
Performance (F1)
Max depth of a tree
2 3 4 5 7 10 None
Number of trees
10
30
100
300
1000

Supplementary Fig. S1. Prediction performances as expressed by the area under the curve (AUC) of the receiver operating characteristic (ROC) curve and F1 score of various machine learning methods with varying hyperparameter sets.

(a, b) Prediction performances of ANN with varying numbers of hidden layers and nodes. The x-axis represents the differing numbers of hidden nodes of each hidden layer. The y-axis shows the (a) AUC of the ROC curve from the sensitivity-specificity plot and (b) F1 score. The performances of the models based on the number of hidden layers are shown as a line. In the case of the F1 score, we depicted the performance of a random prediction and its 95% confidence interval. ANN models with 60 hidden nodes and 5–10 hidden layers show a higher F1 score than the 95% confidence interval of the random prediction performance.

(c, d) Prediction performances of ANN with varying numbers of hidden layers, hidden nodes, and learning rates. The x-axis represents the differing numbers of hidden nodes of each hidden layer. The y-axis shows the (c) AUC of the ROC curve from the sensitivity-specificity plot and (d) F1 score. The performances of the models from each learning rate are depicted as a line. In general, a simple model with a smaller number of nodes favors a high learning rate, whereas a complex model with a larger number of nodes achieves a better performance with a low learning rate. For the 60 hidden nodes used in our best model, the best performance over the other models achieved a value of 0.00003.

(e, f) Prediction performances of XGBoost with varying maximum tree depths and learning rates. The x-axis represents the varying maximum tree depths for each decision tree. The y-axis shows the (e) AUC of the ROC curve from the sensitivity-specificity plot and (f) F1 score. The performances of the models based on the learning rate are shown as a line. The XGBoost model with a maximum tree depth of nine and a learning rate of 0.1 shows a higher F1 score than the 95% confidence interval of the random prediction performance.

(g, h) Prediction performances of XGBoost with varying maximum tree depths, learning rates, and gamma levels. The x-axis represents the differing maximum depths of decision trees. The y-axis shows the (g) AUC

of the ROC curve from the sensitivity-specificity plot and (h) F1 score. The performances of the models from each learning rate are depicted as a line. With a maximum tree depth of nine and a learning rate of 0.1, gamma = 1 shows the best performance.

(i, j) Prediction performances of RF with varying maximum tree depths and numbers of trees. The x-axis represents the differing maximum depths of decision trees. The y-axis shows the (i) AUC of the ROC curve from the sensitivity–specificity plot and (j) F1 score. The performances of the models based on the numbers of trees are depicted as a line. Only the RF model with no limit to the maximum depth of a tree and 30 for number of trees shows a higher F1 score than the 95% confidence interval of a random prediction performance.

(k, l) Prediction performances of RF with varying maximum depths of a tree, number of trees, minimum sample split, and minimum leaf samples. The x-axis represents the differing maximum depths of decision trees. The y-axis shows the AUC of the (k) ROC curve from the sensitivity-specificity plot and (l) F1 score. The performances of the models based on each number of trees are depicted as a line. With no limit to the maximum depth of a tree and 30 for number of trees, a higher minimum sample split shows a higher AUC of the ROC curve but a lower F1 score. The minimum number of leaf samples does not show a consistent variation with the other varying numbers. Because the F1 scores are changed more than the AUC of the ROC curve and because the default minimum sample split for RF is one, we selected this value as the minimum sample split in the following analysis.

Supplementary Fig. S2

a

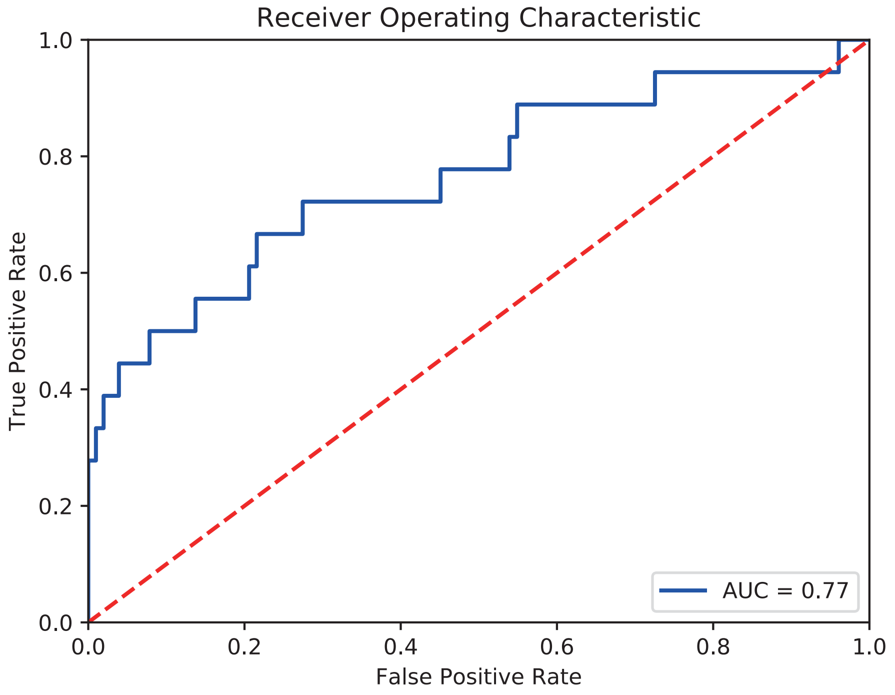

b

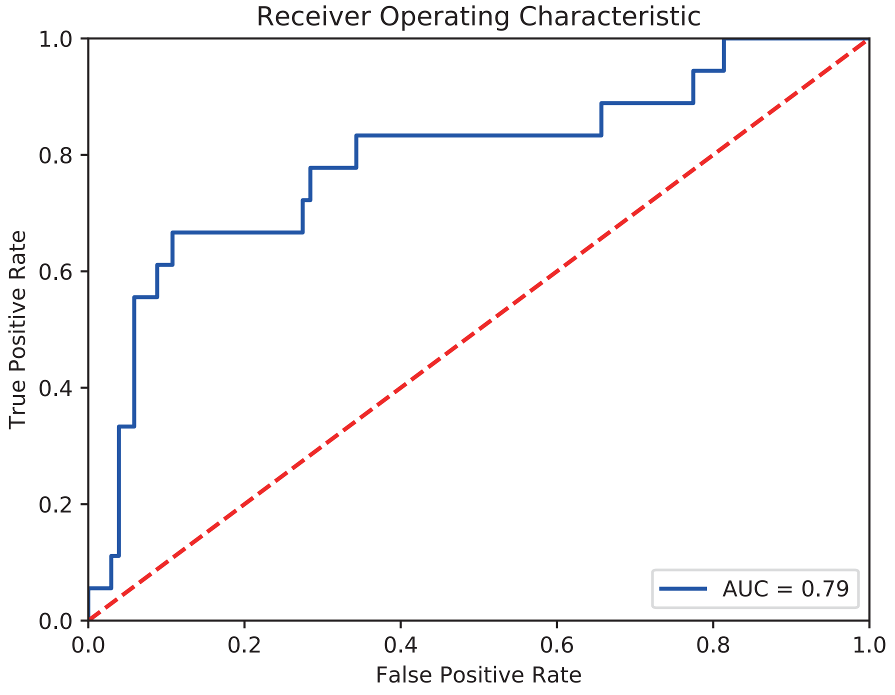

Supplementary Fig. S2. ROC curve of the sensitivity analysis with differed methods for dividing the test dataset. (a) Performance of the model without using cross-validation ($p = 0.766$). (b) Performance of the model without using cross-validation and tested using the independent dataset ($p = 0.791$).

Supplementary Fig. S3

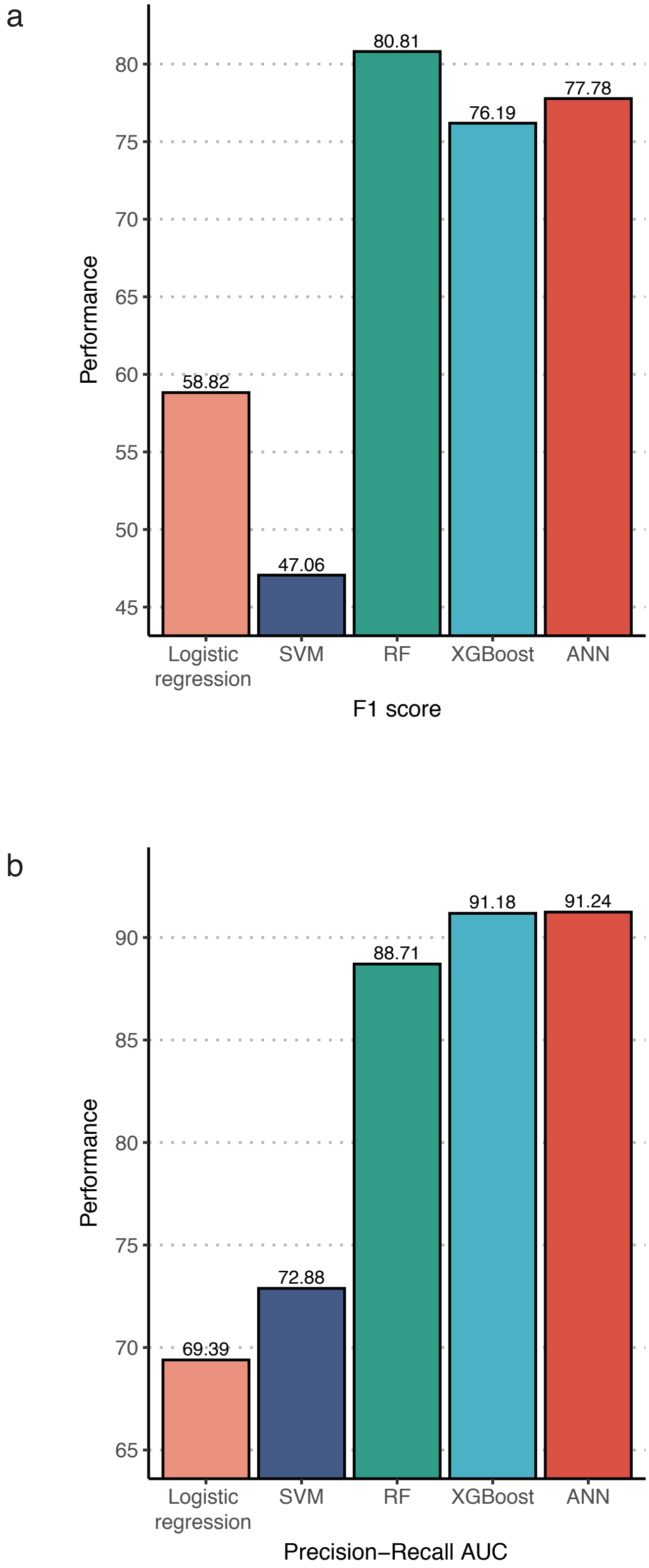

Supplementary Fig. S3. F1 score and AUC of the precision-recall curve with balanced test dataset. (a) F1 score, (b) AUC of the ROC curve.

Supplementary Fig. S4

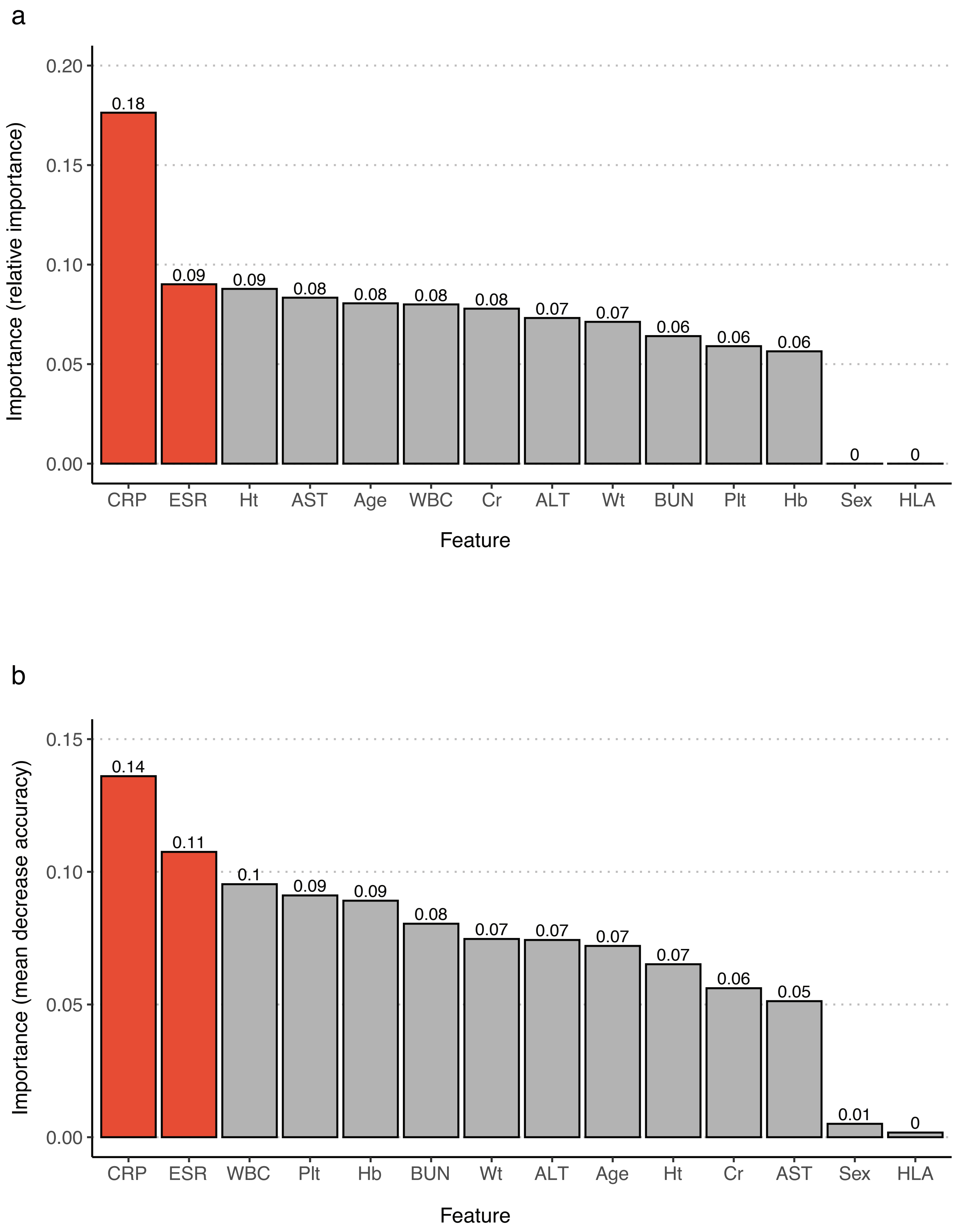

Supplementary Fig. S4. The results of a feature importance analysis from the model trained using (a) XGBoost and (b) RF models. The x-axis shows the clinical variable inputs. The y-axis represents the feature importance score calculated based on (a) the relative importance (b) and the mean decrease in accuracy. The two most important features from the feature importance analysis with the ANN model are highlighted in red.
